# Supplementary material for: GPX2 predicts recurrence-free survival and triggers the Wnt/β-catenin/EMT pathway in prostate cancer
Source: PeerJ. 2022 Oct 25;10:e14263. doi: 10.7717/peerj.14263 (PMC9615941; doi:10.7717/peerj.14263)
Supplement: Supplemental Information 6 [file peerj-10-14263-s006.docx]

Table S1 General information of clinical samples

| samples | Age(year) | Gleason score | tissue type |
| --- | --- | --- | --- |
| 1 | 73 | 3+4=7 | tumour |
| 2 | 77 | 3+3=6 | tumour |
| 3 | 68 | 3+3=6 | tumour |
| 4 | 66 | 4+3=7 | tumour |
| 5 | 71 | 3+4=7 | tumour |
| 6 | 80 | 3+3=6 | tumour |
| 7 | 85 | 4+3=7 | tumour |
| 8 | 69 | 4+3=7 | tumour |
| 9 | 73 | 3+4=7 | tumour |
| 10 | 75 | 3+3=6 | tumour |
| 11 | 67 | 4+4=8 | tumour |
| 12 | 73 | 5+4=9 | tumour |
| 13 | 71 | 5+4=9 | tumour |
| 14 | 70 | 4+5=9 | tumour |
| 15 | 82 | 5+5=10 | tumour |
| 16 | 65 | 5+5=10 | tumour |
| 17 | 69 | 3+5=8 | tumour |
| 18 | 66 | 4+4=8 | tumour |
| 19 | 71 | 5+5=10 | tumour |
| 20 | 65 | 5+4=9 | tumour |
| 21 | 81 | NA | normal |
| 22 | 72 | NA | normal |
| 23 | 64 | NA | normal |
| 24 | 74 | NA | normal |
| 25 | 66 | NA | normal |
| 26 | 82 | NA | normal |
| 27 | 76 | NA | normal |
| 28 | 63 | NA | normal |
| 29 | 65 | NA | normal |
| 30 | 57 | NA | normal |
